# Unveiling the Hidden Culprit: A Case of Bile Leakage Post-Cholecystectomy Caused by a Luschka Duct

**DOI:** 10.5334/jbsr.3233

**Published:** 2023-08-11

**Authors:** Sébastien Selleslag, Mathieu Vandeputte, Marie-Sofie Walgraeve

**Affiliations:** 1 AZ St. Jan Brugge, KU Leuven, BE; 2 AZ St. Jan Brugge, BE

**Keywords:** subvesical bile duct, Luschka duct, subvesicular duct, cholecystectomy, bile duct, bile leak, MRI, laparoscopy

## Abstract

**Teaching Point:** Recognize anatomical bile duct anomalies as a potential etiology of bile leakage post-cholecystectomy, and emphasize the importance of adequate radiological evaluation for correct management.

## Case History

**Figure 1:**
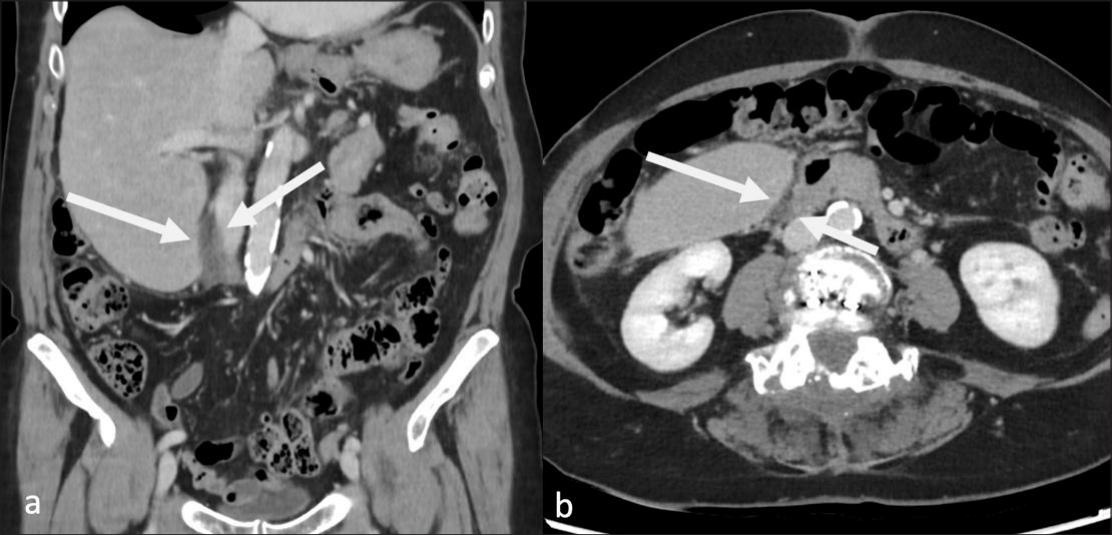


A 74-year-old female patient presented with severe right hypochondrial pain and nausea four days after laparoscopic cholecystectomy. Vital parameters were within normal limits. Physical examination revealed diffuse abdominal tenderness. Laboratory analysis was normal, except for a mild hyperlactatemia (3.3 mmol/L). In order to rule out postoperative bleeding, contrast-enhanced computed tomography (CT) was performed, which revealed a minimal amount of intra-abdominal free fluid at the resection zone of the gallbladder (Figure 1), but no arterial hemorrhage. The patient was subsequently admitted for observation.

**Figure 2:**
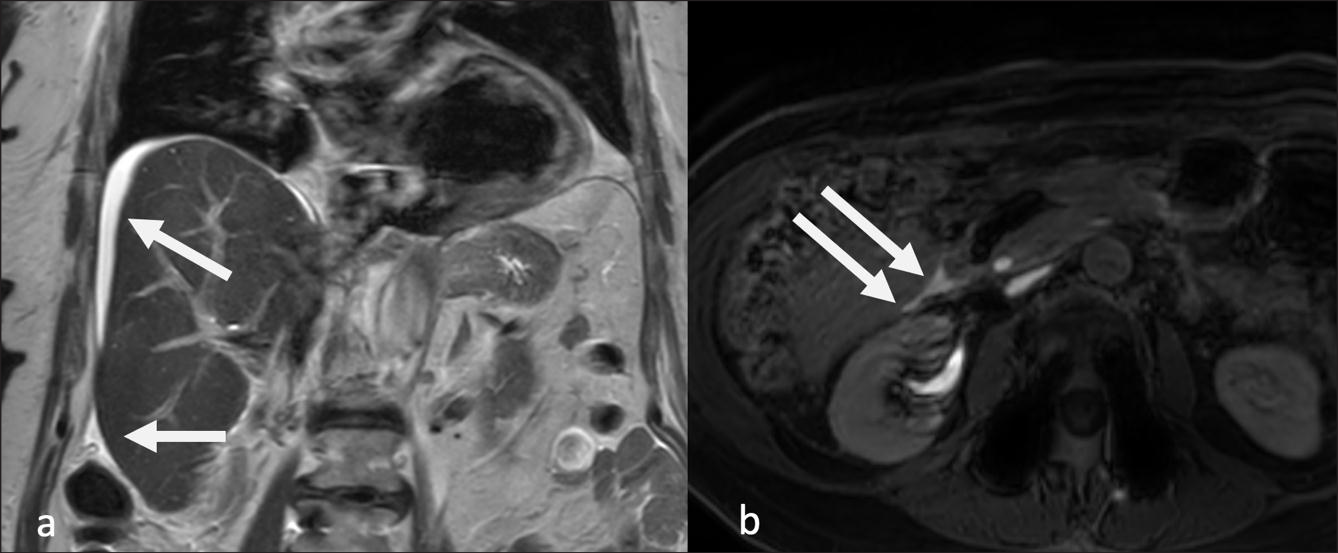


Biochemical re-evaluation demonstrated an increasing white blood cell count (13.6 × 10 ^9^/L), elevated CRP levels (150 mg/L), and persistent, difficult-to-control pain. Consequently, an abdominal magnetic resonance imaging (MRI) scan was performed to exclude the possibility of residual choledocholithiasis. Intravenous Gadobenate dimeglumine (MultiHance, Bracco Imaging S.p.A., Milan, Italy), a hepatobiliary contrast agent, was administered for contrast enhancement. The MRI images revealed a significantly increased amount of intra-abdominal free fluid, especially perihepatically (Figure 2a), but initially no other abnormalities. However, a delayed T1-weighted sequence 2.5 hours after IV contrast administration demonstrated evident contrast leakage in the free fluid, most pronounced at the hepatic hilum (Figure 2b), which led to the diagnosis of bile leakage.

**Figure 3:**
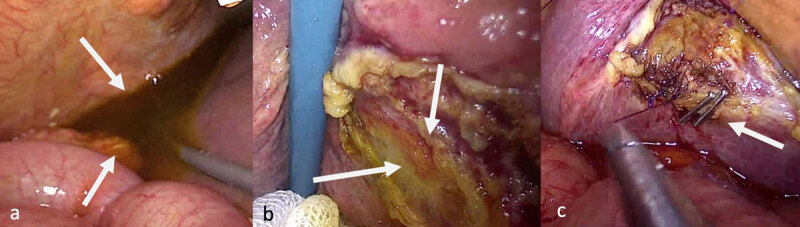


A laparoscopic exploration was performed, confirming the presence of intra-abdominal biliary fluid (Figure 3a). Inspection of the gallbladder bed revealed slowly oozing bile from an accessory subvesicular bile duct, also known as bile duct of Luschka (Figure 3b). The duct was clipped and sutured (Figure 3c). The biliary fluid was aspirated, and the peritoneal cavity was rinsed. The patient’s condition improved, and she was discharged three days later.

## Comments

Anatomical variants of the bile duct system are frequently encountered. Four types have been described: accessory subvesicular or Luschka ducts, (chole)cystohepatic ducts, segmental or sectorial bile duct variants, and aberrant bile ducts. Luschka ducts, more correctly known as accessory subvesicular ducts, represent the most common variant, with reported incidences ranging from 12% to 50%. These ducts originate from the right hepatic lobe and course along the gallbladder fossa to drain into the main bile ducts. Due to their small diameter (approximately 2 mm) and inconspicuous nature, Luschka ducts can easily go unnoticed and become injured during surgical procedures. Preoperative identification is rare, with most diagnoses being made postoperatively, typically due to bile leakage and associated complications [1]. Although ultrasound and CT can serve as imaging modalities, MRI is considered the optimal non-invasive imaging technique for exploration of biliary tract pathology. Particularly delayed images performed 90 minutes or longer after hepatobiliary contrast administration have high sensitivity and specificity in confirming bile leakage, as demonstrated in this case.
